# Transcriptome profiling of intrahepatocytic *Plasmodium* and their host hepatocytes based on the infection phase and the zonation of the liver

**DOI:** 10.3389/fgene.2025.1548487

**Published:** 2025-04-07

**Authors:** Zhuning Mo, Yuhong Chen, Yujuan Qin, Jian Song

**Affiliations:** 1 Institute of Cardiovascular Sciences, Guangxi Academy of Medical Sciences, Nanning, China; 2 Department of Blood Transfusion, The People’s Hospital of Guangxi Zhuang Autonomous Region, Nanning, China; 3 Department of Radiation Oncology, Renji Hospital, School of Medicine, Shanghai Jiao Tong University, Shanghai, China

**Keywords:** *Plasmodium*, intraerythrocytic stage, dual transcriptome, spatiotemporal analyses, contrast between *in vivo* and *in vitro*

## Abstract

Intrahepatocytic development is a key stage in human *Plasmodium* infection, in which sporozoites replicate and transform into merozoites. Due to technological limitations, however, previous gene expression studies on malaria parasite liver infection were mostly conducted *in vitro*. In order to bridge these gaps, our current study compared the gene expression of *in vitro*-infected parasites at different time points with that of *in vivo*-infected parasites and revealed distinct patterns between parasite subpopulations *in vitro* and *in vivo*. A joint investigation of the *Plasmodium* transcriptome and their host transcriptome was carried out to confer a comprehensive analysis of gene expression in the liver stage of *Plasmodium* infection *in vivo*, which is similar to the disease setting, and therefore deepen our understanding of parasite and host transcriptional dynamics during intrahepatocytic infection.

## Graphical Abstract

**Figure:**
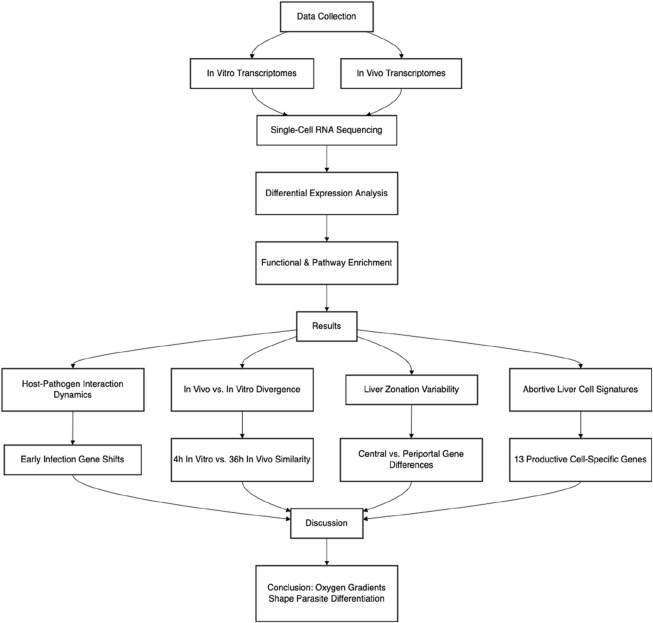

## 1 Introduction

Malaria is a vector-borne infectious disease caused by *Plasmodium* parasites that have complex life cycles with various life stages in mosquito vectors and human hosts. Key stages in the human host include the liver stage and the intraerythrocytic development (IDE) period (Vaughan and Kappe, 2017). After transmission by mosquitoes, sporozoites enter the bloodstream and migrate to the liver. Within liver cells, sporozoites transform into merozoites, replicate extensively, and release these merozoites into the bloodstream (Lopes da Silva et al., 2012). In the liver stage, *Plasmodium* employ various mechanisms to escape the host immune reactions, taking advantage of the immunoprivileged nature of liver cells, which limits immune surveillance (Little et al., 2021; Real et al., 2021). Parasites modulate host cell signals, suppressing the activation of immune responses, including interference with interferon signaling and other crucial immune activation pathways (Liehl et al., 2014; Inacio et al., 2015; Ribot et al., 2019). Parasite proteins may also disrupt antigen presentation pathways, reducing immune cell visibility of infected liver cells (Hodgson et al., 2019; Mancio-Silva et al., 2022). Parasites strive to minimize antigen expression recognizable by the immune system, evading detection (Toro-Moreno et al., 2020; Hirako et al., 2022). Understanding these evasion mechanisms is crucial for developing strategies targeting the liver stage and preventing infection. Therefore, it is important not only to study the transformation of *Plasmodium* in the liver stage but also to know the ways in which liver cells respond to the parasites.

Understanding the complex life cycle of *Plasmodium*, especially its liver stage, is crucial for developing effective preventive and therapeutic strategies. With the advent of molecular biology techniques, gene expression studies on the *Plasmodium* liver stage emerged. Although these studies provided valuable information about the heterogeneity of host cells during infection, they often focused solely on the host side and did not jointly investigate the transcriptomes of both the parasite and the host in a comprehensive manner. In addition, as most of the previous molecular mechanism studies were based on the *in vitro* infection of the parasite because of its availability, it is important to integrate the *in vitro* information into the emerging *in vivo* studies (Howick et al., 2019).

Recent studies using the combination of single-cell sequencing and dual RNA sequencing (RNA-seq) allowed the investigation of *in vivo* stages (Afriat et al., 2022; Yang et al., 2023; Zanghi et al., 2023). In order to bridge these gaps, we jointly investigated the transcriptome profiles of intrahepatocytic *Plasmodium* and their host hepatocytes by using deposited RNA-seq data and single-cell sequencing data. To map these recent results to the vast amount of information from previous studies, we established a method based on gene set enrichment analysis (GSEA) and compared the gene expression of *in vitro*-infected parasites at different time points with that of *in vivo*-infected parasites, which revealed distinct patterns. This approach allows us to gain a more comprehensive understanding of the infection dynamics and gene expression patterns during the *Plasmodium* liver stage. It can potentially uncover novel molecular targets for anti-malarial drugs and provide new insights into the complex interactions between the parasite and the host at the molecular level.

## 2 Methods

### 2.1 Data sources

The bulk transcriptome data were derived from the previous datasets (Toro-Moreno et al., 2020; Caldelari et al., 2019; LaMonte et al., 2019), comprising multiple *Plasmodium* datasets collected at various times after infection *in vitro*. Furthermore, data for single-cell transcriptional dynamics during different phases of *Plasmodium in vitro* were sourced via the interactive Malaria Cell Atlas website (www.sanger.ac.uk/science/tools/mca/mca/). Single-cell transcriptome information on *Plasmodium* and their liver cell host at different infection times was collected from GEO databases (GSE181725).

### 2.2 Single-cell RNA sequencing (scRNA-seq)

The Seurat (version 4.2) package was used to analyze the scRNA-seq data. Cells with less than 300 or with more than 20% mitochondrial genes were excluded. After filtering, the data were normalized using the LogNormalize method. Uniform Manifold Approximation and Projection (UMAP) was established utilizing the primary components. The cluster analysis of single-cell data was performed using the graph-based clustering method in Seurat. The resolution of the FindClusters feature was set to 0.1.

### 2.3 Differential expression analyses

Differential expression analyses between the two groups were conducted using the limma package, and the filter condition for differentially expressed genes (DEGs) was set to the adjusted p-value < 0.05. The heatmap plot was built to visualize DEGs using the heatmap package. For the generation of volcano plots, we used ggplot2 in the R software package.

Subsequently, triwise plots and rose diagrams were generated with the log-transformed data using the triwise R software package (van de Laar et al., 2016).

### 2.4 Functional and pathway enrichment analyses

The clusterProfiler package served to target genes for functional and pathway enrichment analyses (Yu et al., 2012). Gene set enrichment analysis (www.gsea-msigdb.org/gsea/index.jsp) was executed for the respective DEGs (transcriptome-defined) to investigate whether these genes were enriched in the compared gene profiles of the *Plasmodium* or host transcriptome. In GSEA, we first ranked all the genes in our dataset based on their differential expression fold change. Then we tested whether the genes in a pre-defined gene set were preferentially located at the top or bottom of this ranked list. The software calculates an enrichment score (ES) for each gene set. A positive ES indicates that the genes in the gene set are upregulated under a particular condition, whereas a negative ES indicates downregulation.

The enrichment levels of the gene signatures determined from 4-h infection by *Plasmodium berghei* were then used to perform hierarchical clustering. Finally, the samples were divided into two directional groups based on the requirement of different time points or *in vitro*/*in vivo* comparison.

Furthermore, Gene Ontology (GO) functional enrichment analyses were performed. The results were visualized using the ggplot2 R software package.

## 3 Results

### 3.1 Host–pathogen interaction dynamics

**FIGURE 1:**
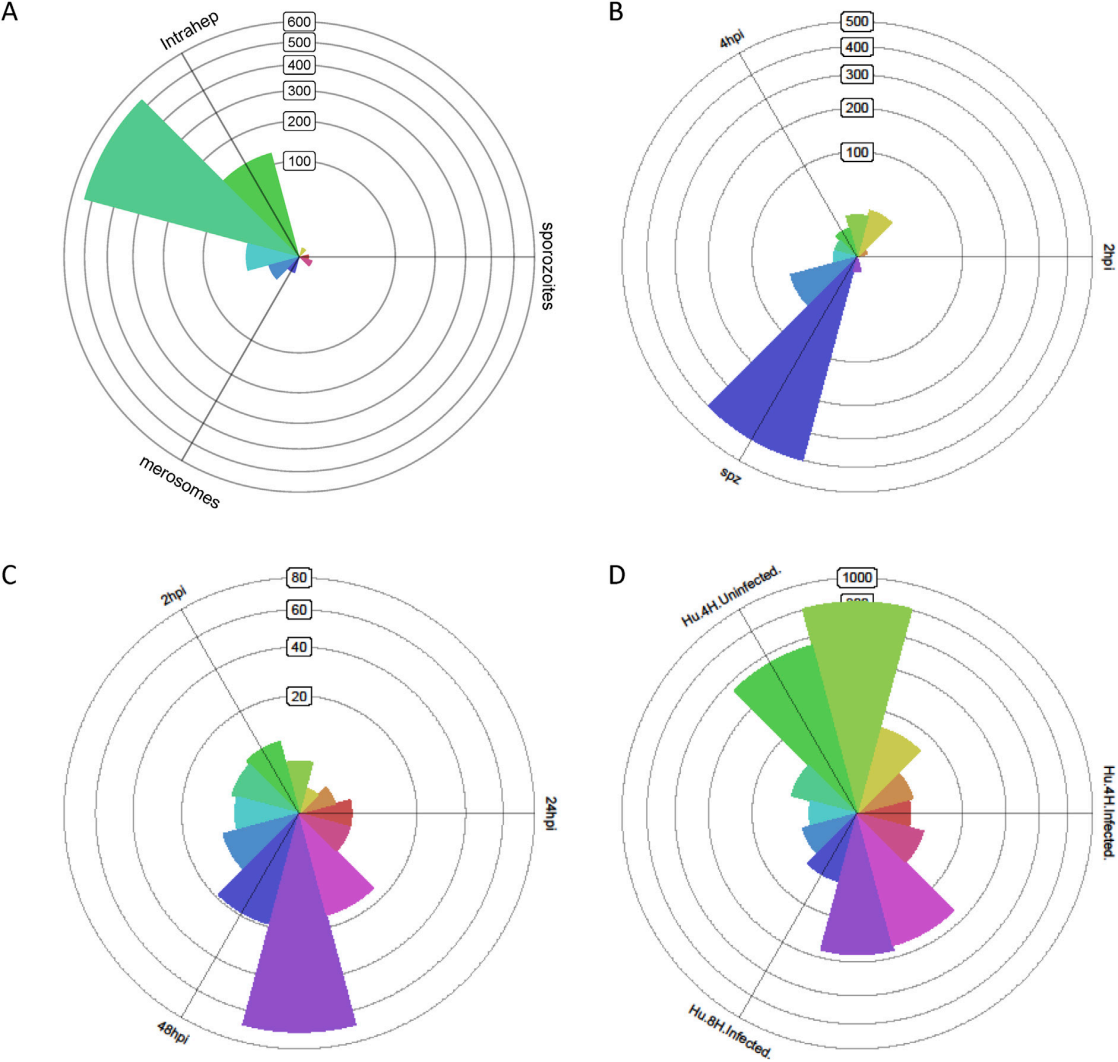
Transcriptome of *Plasmodium* that infected liver cells, and the infected liver cells demonstrate distinct transcriptional profiles at different stages. **(A)** Triwise analysis shows that the transcriptome of *Plasmodium berghei* that infected liver cells is distinctly different from that of sporozoites and merozoites. **(B)** Triwise analysis shows that the transcriptome of sporozoites is distinctly different from that of *Plasmodium* that infected liver cells. **(C)** Triwise analysis shows that the transcriptome profile of *Plasmodium berghei* at the late-stage liver infection is markedly different from the transcriptome profile of those at the early-stage liver infection. **(D)** The transcriptome of hepatocytes infected with *Plasmodium berghei* is distinct from that of hepatocytes not infected with *Plasmodium berghei*.

When comparing the gene expression profile of *Plasmodium* in three major states before and after malaria parasite liver infection, distinct differences were observed. We identified 713 DEGs between sporozoites and *Plasmodium berghei* that infected liver cells, with a fold change of more than 10 and all having an adjusted p-value < 0.001. The roseplot in Figure 1A indicates the major upregulation of *Plasmodium* genes toward the direction of the intrahepatocytic phase when compared with those in sporozoites and merozoites (Figure 1A). This indicates that the gene expression of *Plasmodium* undergoes significant changes during its development within liver cells. The differences in gene expression profiles are likely related to the different functions and survival strategies of the parasite at these different life stages. For example, sporozoites are in the stage of invading liver cells, sporozoite replication within liver cells results in merozoite release, and the *Plasmodium* that infected liver cells are in an intermediate developmental stage.

Compared to early stages of liver infection, gene expression of parasites differed markedly before entering liver cells. The 502 most differentially expressed genes at the early stage of liver infection demonstrated a 10-fold upregulation (p < 0.001) compared to pre-infection sporozoites. The roseplot in Figure 1B indicates the major upregulation of the *Plasmodium* genes toward the direction of the sporozoite phase when compared with the intrahepatocytic phase at 2 h and 4 h of infection (Figure 1B). This further emphasizes the dramatic transformation in the parasite’s gene expression once it enters the liver cells. Sporozoites have specific genes that involve in migration and invasion, whereas *Plasmodium* in the infected liver express genes related to replication and interaction with the host cell.

As the infection progresses, the parasite modifies its gene expression to adapt to the host environment, evade the immune system, and continue its replication. There were 149 significantly differentially expressed *Plasmodium* genes between infected and non-infected hepatocytes, showing a 10-fold increase in infected cells (p-value < 0.001). The roseplot in Figure 1C indicates the major upregulation of *Plasmodium* genes toward the direction of the intrahepatocytic phase at 48 h of infection when compared with the intrahepatocytic phase at 2 h and 24 h of infection (Figure 1C). In contrast, there was some similarity in the transcriptome of hepatocytes after 4 h of infection (Figure 1C).

On the other hand, dramatic differences were also found in the transcriptome of host cells. To study the host–pathogen interaction during malaria parasite liver infection, a dual RNA sequencing study was conducted, comparing the parasites and their host at different stages. Infected liver cells demonstrated 2,666 genes with a fold change of 10 and p-value <0.001 compared to non-infected hepatocytes (Figure 1D). This indicates that the infection has a significant impact on the host hepatocyte’s gene expression, altering its normal functions. The infected hepatocytes may respond to the parasite by changing their metabolic pathways, immune-related gene expression, and cell-cycle regulation.

### 3.2 *In vivo* vs. *in vitro* transcriptional divergence

**FIGURE 2:**
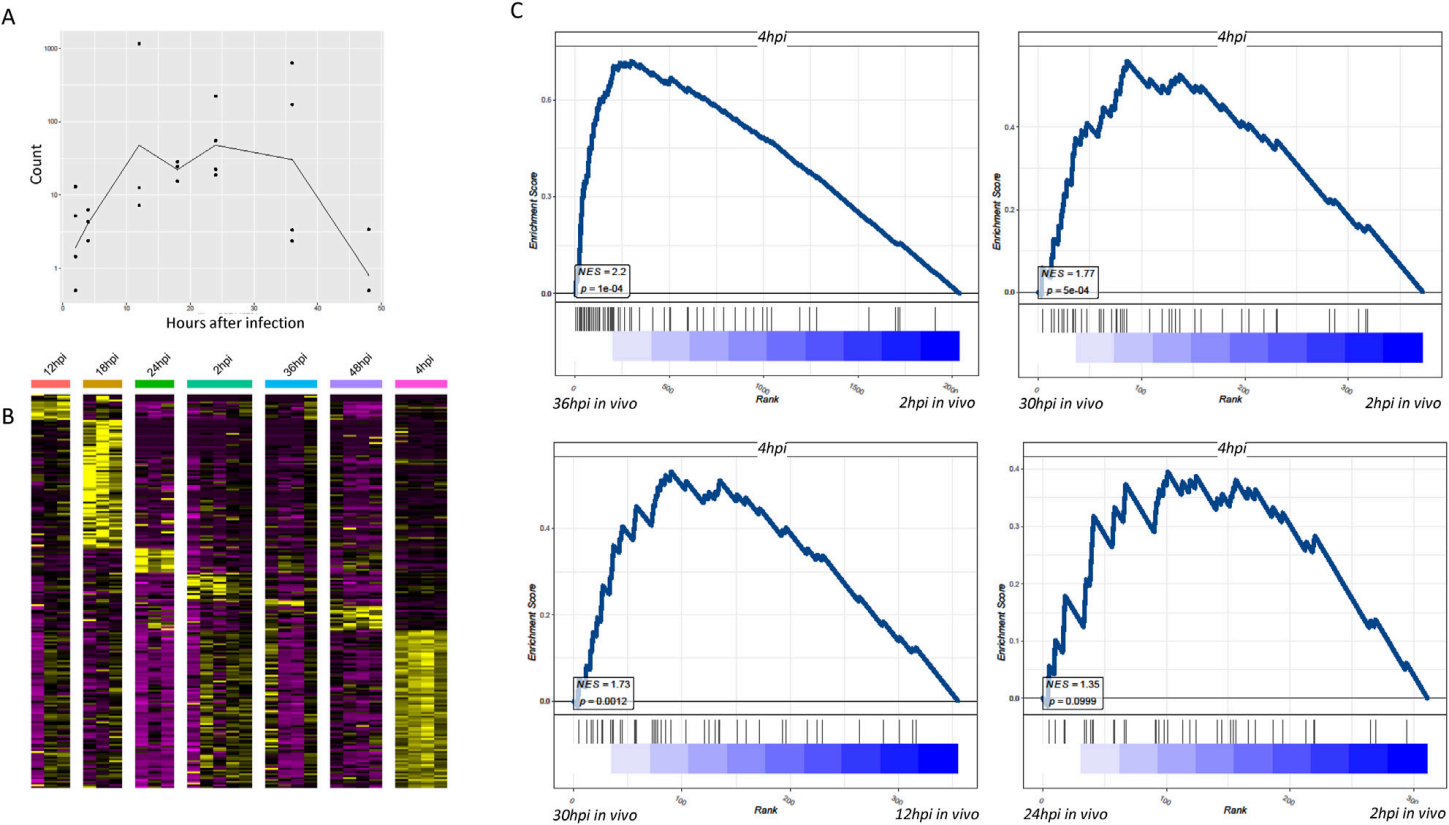
Dual RNA sequencing findings indicate that the transcriptomes of *Plasmodium* that infected the liver and their host cells are distinctly different from that of *Plasmodium* infected *in vitro*. **(A)** Line graph showing temporal profiles of gene expression in parasites infected *in vitro*. The curves in the figure represent the average of total counts of DEGs with adjusted p-values <0.01. DEGs are determined across different time points and samples, which have therefore been normalized. **(B)** Heatmap showing that relative to other time periods, *Plasmodium* demonstrate the most specific gene expression in hepatocytes 4 h after *in vitro* infection. Time hours in the figure represent the characteristic genes of *Plasmodium* after the respective time in the liver cells. Compared to other time hours, 4-h infection confers the most pronounced number of DEGs. **(C)** Using the transcriptome profile of *in vitro*-infected *Plasmodium* at 4 h, we compared the transcriptomes of hepatocytes infected at different times *in vivo*. Both normalized enrichment score and p-values of GSEA scoring indicated the similarity in the *Plasmodium* gene profiling between the *in vivo* group and 4-h *in vitro* infection group.

The combination of single-cell sequencing and dual RNA sequencing allowed the investigation of *in vivo* stages. Comparing gene expression of *in vitro*-infected parasites at different time points with that of *in vivo*-infected parasites revealed distinct patterns. Notably, the *in vitro*-infected parasites exhibited a plateau in gene expression at 4 h, followed by a significant decline after 48 h, with the most specific gene expression observed at 4 h after infection (Figure 2A). This pattern indicates that the *in vitro* culture conditions led to a unique gene expression dynamic, which may not fully represent the *in vivo* situation. The specific gene expression at 4 h might be related to the initial adaptation of the parasite to the *in vitro* environment. *In vitro* parasites exhibit transient gene activation, whereas *in vivo* parasites maintain sustained expression (Figure 2A). This is probably because of oxygen gradients and host immune evasion mechanisms (HIF-1α signaling in pericentral zones), which could act as drivers of divergence between *in vitro* and *in vivo* environments.

When comparing *in vivo* infected liver cells at 36 h, their gene transcription characteristics were more identical to those of *in vitro*-infected parasites at 4 h, indicating significant differences between *in vitro* cell culture and *in vivo* infection (Figure 2B). This further supports the finding in Figure 2A and suggests that 4 h is a critical time point for the parasite’s gene expression *in vitro*. It could be a time when the parasite is actively interacting with the *in vitro* cultured hepatocytes and adjusting its gene expression accordingly.

Using the transcriptome profile of 4-h *in vitro*-infected *Plasmodium*, we compared the transcriptomes of hepatocytes infected at different times *in vivo*. Both normalized enrichment score (NES) and p-values of GSEA scoring indicated the similarity of *Plasmodium* gene profiling between the *in vivo* group and the 4-h *in vitro* infection group (Figure 2C). The analysis of transcriptional profile differences between *in vitro* and *in vivo* was challenging before 24 h of *in vivo* infection, suggesting different transformation times for parasites within liver cells *in vivo*. This implies that the transformation time of parasites within liver cells *in vivo* is different from that in the *in vitro* environment, and it takes time for the differences in gene expression to become apparent. The *in vivo* environment, with its complex immune system and physiological factors, may influence the parasite’s gene expression in a way that is distinct from that in the *in vitro* culture.

### 3.3 Regional variability in liver zonation and infection outcomes

**FIGURE 3:**
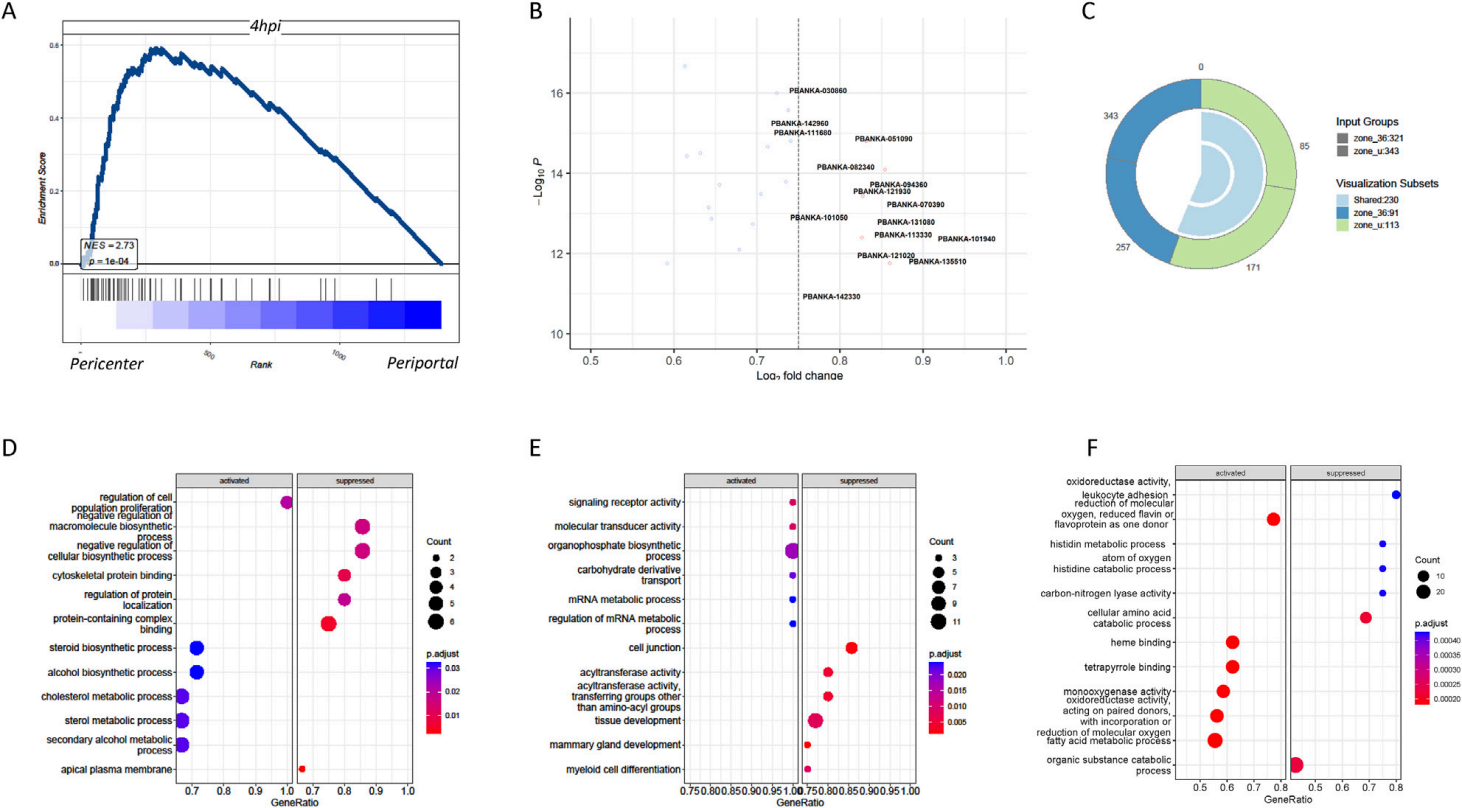
Significant differential transcriptome between *Plasmodium* in hepatocytes around the central vein and the portal vein as well as their host transcriptome. **(A)** GSEA showed that compared to the periportal infection, the gene expression of pericentrally infected *Plasmodium* significantly resembled the gene expression of those infected *in vitro* after 4 h. **(B)** Volcano graph comparing the gene expression of *Plasmodium* in the pericentral and periportal regions of the liver. **(C)** Venn diagram of host DEGs in pericentral (PC) versus periportal (PP) zones. Zone_u: 250 unique DEGs in uninfected cells; zone_36: 180 unique DEGs in infected cells; overlap: 45 shared genes. **(D)** GO enrichment analyses showed the host differential genes specific to no infection in pericentric versus periportal hepatocytes. **(E)** GO enrichment analyses showed the host differential genes specific to 36-h infection *in vivo* in pericentral versus periportal hepatocytes. **(F)** GO enrichment analyses showed the shared host differential genes by both no infection and 36-h infection in pericentral versus periportal hepatocytes.

Apart from the timeliness of malaria parasite infection in liver cells, the complex liver environment may influence the differentiation status of the parasites. Studies have shown significant differences in gene expression between parasites infecting the cells in the pericentral region and those around the periportal region of the liver (Afriat et al., 2022). The central region had higher infection abundance, whereas the survival rate of parasites infecting liver cells around the portal vein was lower, possibly related to uneven oxygen distribution in liver lobules (Ben-Moshe and Itzkovitz, 2019). We found that although parasite-infected liver cells exhibited a distinct gene profiling based on their infected time (Supplementary Figure S1A), almost all the temporal clusters contain clear spatial segregation based on the expression of periportally or pericentrally zonated genes (Supplementary Figure S1B–D). GSEA indicated that parasites infecting the cells in the pericentral region of the liver closely resembled parasites that infected *in vitro* at 4 h (Figure 3A), suggesting that *in vitro* culture primarily simulates parasites infecting the cells in the central region of the liver in an oxygen-rich state. However, further research is needed to understand the *in vitro* model of parasites infecting liver cells around the portal vein. The genes associated with parasites infecting the cells in the pericentral region of the liver at 36 h *in vivo* were mainly related to synthesis (Figure 3B). These differential genes can provide insights into the different adaptation mechanisms of the parasite while infecting cells in different regions of the liver. For example, genes related to metabolism, immune evasion, and cell adhesion may be differentially expressed to cope with the different microenvironments in the pericentral and periportal regions. To study the host cells infected in the pericentral and portal vein regions, differential genes between the infected cells in the pericentral and portal regions of the liver, including uninfected and 36-h *in vivo* infected cells, were compared. Venn diagrams delineated unique differential genes for uninfected and 36-h *in vivo* infected cells (Figure 3C), indicating detoxification and transformation functions for uninfected cells and associations with alcohol and cholesterol metabolism for 36-h *in vivo* infected cells (Figures 3D–F). The common genes were associated with oxygen levels.

### 3.4 Significance of abortive liver cells

**FIGURE 4:**
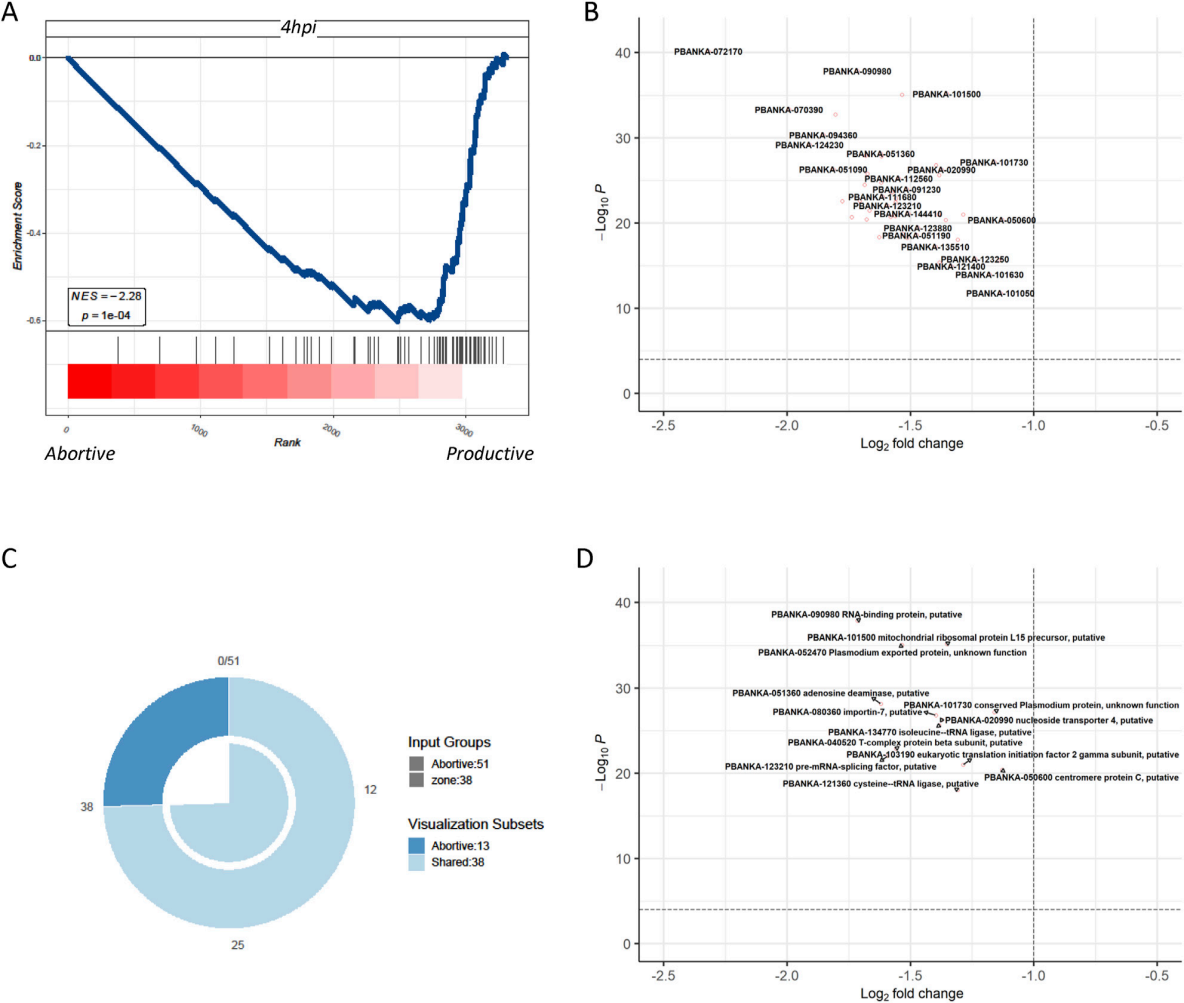
Comparison of the differential genes of *Plasmodium* in abortive hepatocytes and in productive cells with the genetic profile of *Plasmodium* after 4 h of *in vitro* infection **(A)** GSEA shows that compared to the transcriptome of *Plasmodium* in abortive hepatocytes, the transcriptome of *Plasmodium* in productive cells significantly resembled the transcriptome of those infected *in vitro* after 4 h. **(B)** Differential genes in Plasmodium in abortive hepatocytes versus productive cells at 4 hours post-infection in *Plasmodium* in abortive hepatocytes versus those in *Plasmodium* in productive cells. **(C)** Venn diagrams define, respectively, the *Plasmodium*-specific genes of the productive cells, *Plasmodium*-specific genes of pericentral infection, and their shared genes. **(D)** Volcano graph showing the 13 related genes that were specific to the productive cells.

The most notable discovery in *in vivo* malaria parasite liver infection was the identification of abortive liver cells, which were not previously observed in *in vitro* experiments. To contrast with *in vitro* cultured parasites, the differential genes of abortive liver cells at 36 h *in vivo* and those of productive cells were compared with the gene features of parasites infected *in vitro* at 4 h. It was found that the gene features of productive cells closely resembled those of parasites infected *in vitro* at 4 h, with genes primarily associated with parasite synthesis (Figure 4A). This suggests the difficulty of simulating abortive liver cell in parasites in the *in vitro* culture. Productive cells, which are more identical to *in vitro*-infected parasites at 4 h, may have a different set of gene expression patterns related to successful replication and development, whereas abortive cells have a distinct gene expression pattern that makes their replication complex *in vitro*. Genes related to DNA replication, protein synthesis, and cell-cycle regulation may be differentially expressed between abortive and productive cells (Figure 4B). Although there is an overlap, productive cells also contain unique genes, indicating that they have specific characteristics that are not fully captured by the pericentral infection model (Figure 4C). The volcano graph shows the 13 related genes that are specific to the productive cells (Figure 4D). These genes are likely to play important roles in the successful development of *Plasmodium* in hepatocytes and may be potential targets for future studies on malaria treatment or prevention. The data not only indicate similarity between the gene features of productive cells and parasites cultured *in vitro*, similar to those infecting cells in the pericentral region of the liver in an oxygen-rich state (Hirako et al., 2022; Ben-Moshe and Itzkovitz, 2019; Ben-Moshe et al., 2019) but also suggest that these are the major genes shared between liver cells in the pericentral and portal regions.

## 4 Discussion

Our data on gene expression during *Plasmodium* liver infection emphasize the importance of the early stages of *Plasmodium* infection. In-depth analyses of single-cell profiles of *Plasmodium*-infected hepatocytes *in vivo* revealed differences between parasite subpopulations *in vitro* and *in vivo*. A joint investigation of the *Plasmodium* transcriptome and their host transcriptome provided a comprehensive analysis of gene expression in the liver stage of *Plasmodium in vivo*, deepening our understanding of parasite and host transcriptional dynamics during liver infection.

To study host–pathogen interactions in *Plasmodium* liver infections, we have taken advantage of the fact that single-cell sequencing technology can compare the transcriptomes of the host and the pathogen in a cell. We performed a dual RNA sequencing study and compared the transcriptome profiles of not only infected hepatocytes at different times but also those at different locations in the physiological and infected states, which were compared with the corresponding cellular pathogen transcriptomes. This comparison allowed us to find that the pathogen transcriptome of pericentric hepatocytes infected with *Plasmodium berghei* was more identical to that of *Plasmodium berghei* infection *in vitro*, which may be related to the oxygen content.

The bile ducts in the hepatic lobules carry bile acids secreted by the hepatocytes outward in a direction opposite to that of blood flow to the portal node. In contrast, blood enters the lobules from the portal node and flows inward through the hepatic blood sinusoids to the draining central vein (Hoehme et al., 2010). Hepatocyte zonation facilitates the allocation of high-energy-consuming tasks to high-oxygen areas and spatial recycling of materials (Ben-Moshe and Itzkovitz, 2019). The host transcriptome profiles of infected pericentric hepatocytes and uninfected pericentric hepatocytes were mostly identical, suggesting the positional conservation of the hepatocyte transcriptome. Interestingly, the host transcriptome after infection not only has a portion of the unique genes but also loses a portion of the site-specific genes of the hepatocyte transcriptome, suggesting that these genes were originally associated with the stress response and that pericentric and portal hepatocytes not only differ in metabolic function but also play different roles related to infection.

Hepatocytes exhibit significant variability in their transcriptome depending on their location along the central axis of the lobular portal vein. This heterogeneity is a consequence of the intrinsic epigenetic features inherent in the partitioning of oxygen, nutrient, and hormone gradients. The study of *Plasmodium* infection in hepatocytes requires systematic characterization of these heterogeneous layers to better understand *Plasmodium* differentiation and liver dysfunction during *Plasmodium* infection.

In summary, the current studies contribute to our understanding of malaria parasite liver infection dynamics, uncovering crucial gene expression patterns and molecular interactions at various stages.

## Data Availability

The original contributions presented in the study are included in the article/Supplementary Material; further inquiries can be directed to the corresponding author.
