# Correction: Transcriptome profiling of intrahepatocytic *Plasmodium* and their host hepatocytes based on the infection phase and the zonation of the liver

**DOI:** 10.3389/fgene.2026.1799315

**Published:** 2026-02-06

**Authors:** Zhuning Mo, Yuhong Chen, Yujuan Qin, Jian Song

**Affiliations:** 1 Institute of Cardiovascular Sciences, Guangxi Academy of Medical Sciences, Nanning, China; 2 Department of Blood Transfusion, The People’s Hospital of Guangxi Zhuang Autonomous Region, Nanning, China; 3 Department of Radiation Oncology, Renji Hospital, School of Medicine, Shanghai Jiao Tong University, Shanghai, China

**Keywords:** *Plasmodium*, intraerythrocytic stage, dual transcriptome, spatiotemporal analyses, contrast between *in vivo* and *in vitro*

An incorrect **Funding statement** was provided. The correct funders are the “Natural Science Foundation of Guangxi Zhuang Autonomous Region” and the “National Natural Science Foundation of China”.

The correct **funding statement** reads: The author(s) declare that financial support was received for the research and/or publication of this article. This study would not be possible without the funding from the Natural Science Foundation of Guangxi Zhuang Autonomous Region (2023GXNSFAA026041) and the National Natural Science Foundation of China (82060036, 32360176).

The original article has been updated.

