# Supplementary figures and images for: Transcriptome profiling of intrahepatocytic Plasmodium and their host hepatocytes based on the infection phase and the zonation of the liver
Source: Front Genet. 2025 Apr 7;16:1548487. doi: 10.3389/fgene.2025.1548487 (PMC12042280; doi:10.3389/fgene.2025.1548487)

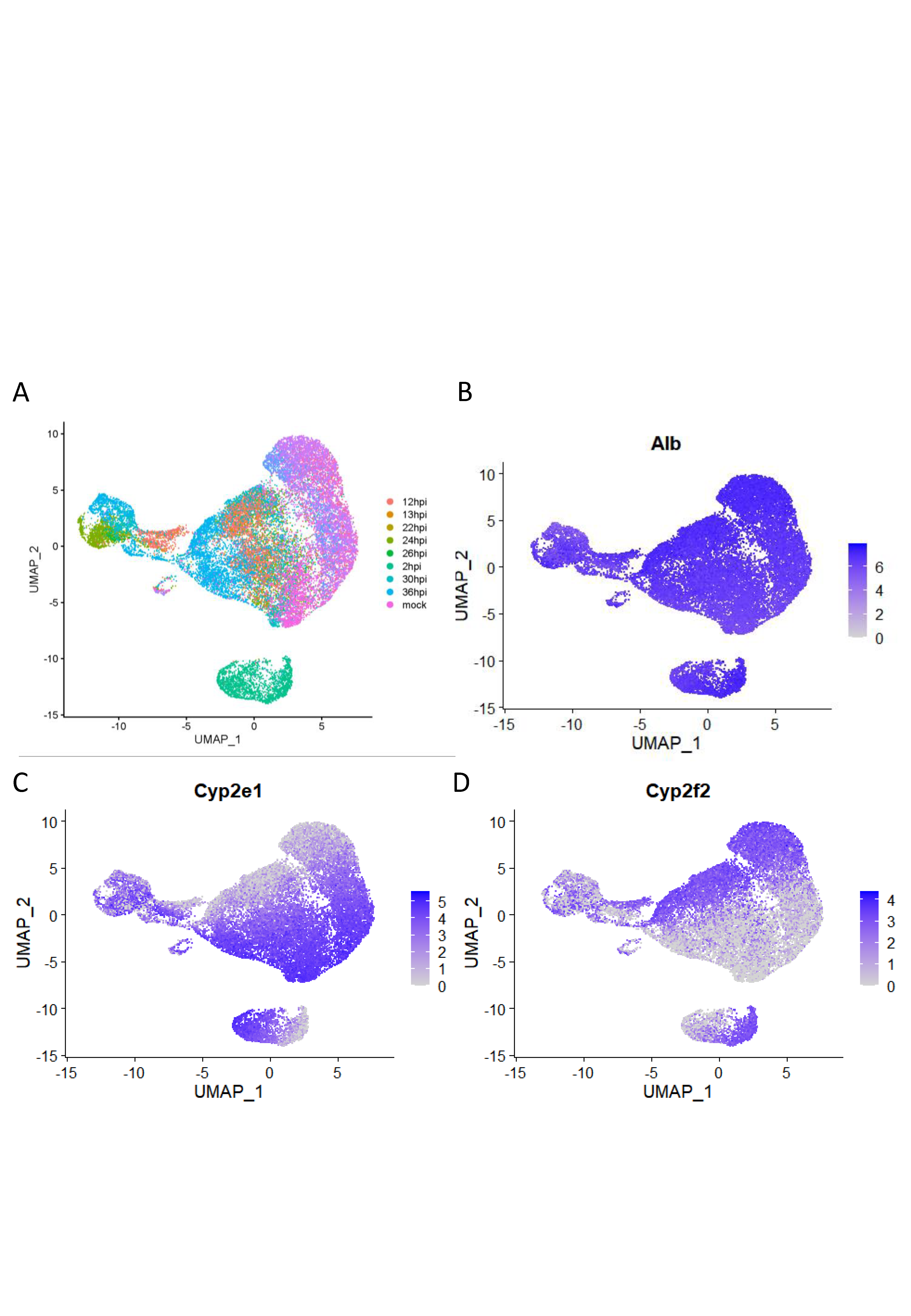

Supplement: Supplementary file 1 [file Image1.tiff]
